# *Potamophylax coronavirus* sp. n. (Trichoptera: Limnephilidae), a new species from Bjeshkët e Nemuna National Park in the Republic of Kosovo, with molecular and ecological notes

**DOI:** 10.3897/BDJ.9.e64486

**Published:** 2021-04-07

**Authors:** Halil Ibrahimi, Astrit Bilalli, Simon Vitecek, Steffen U. Pauls, Felicitas Erzinger, Agim Gashi, Linda Grapci Kotori, Donard Geci, Milaim Musliu, Edison Kasumaj

**Affiliations:** 1 University of Prishtina "Hasan Prishtina", Faculty of Mathematics and Natural Sciences, Department of Biology, Eqrem Çabej nn., Prishtina, Kosovo University of Prishtina "Hasan Prishtina", Faculty of Mathematics and Natural Sciences, Department of Biology, Eqrem Çabej nn. Prishtina Kosovo; 2 University of Peja "Haxhi Zeka", Faculty of Agribusiness, UÇK str. nn., Peja, Kosovo University of Peja "Haxhi Zeka", Faculty of Agribusiness, UÇK str. nn. Peja Kosovo; 3 University of Natural Resources and Life Sciences, Vienna Institute of Hydrobiology and Aquatic Ecosystem Management (IHG) Gregor-Mendel-Straße 33, 1180, Vienna, Austria University of Natural Resources and Life Sciences, Vienna Institute of Hydrobiology and Aquatic Ecosystem Management (IHG) Gregor-Mendel-Straße 33 1180, Vienna Austria; 4 WasserCluster Lunz – Biologische Station, Dr. Carl Kupelwieserpromenade 5, 3293, Lunz am See, Austria WasserCluster Lunz – Biologische Station, Dr. Carl Kupelwieserpromenade 5 3293, Lunz am See Austria; 5 University of Vienna, Department of Functional and Evolutionary Ecology, Althanstraße 14, 1090, Vienna, Austria University of Vienna, Department of Functional and Evolutionary Ecology, Althanstraße 14 1090, Vienna Austria; 6 Institute of Insect Biotechnology, Justus-Liebig-University Gießen, Heinrich-Buff-Ring 26, 35392, Gießen, Germany Institute of Insect Biotechnology, Justus-Liebig-University Gießen, Heinrich-Buff-Ring 26, 35392 Gießen Germany; 7 Senckenberg Research Institute and Natural History Museum Frankfurt, Senckenberganlage 25, 60388, Frankfurt a. M., Germany Senckenberg Research Institute and Natural History Museum Frankfurt, Senckenberganlage 25, 60388 Frankfurt a. M. Germany; 8 Senckenberg Biodiversity and Climate Research Centre (BiK-F), Senckenberganlage 25, 60388, Frankfurt a. M., Germany Senckenberg Biodiversity and Climate Research Centre (BiK-F), Senckenberganlage 25, 60388 Frankfurt a. M. Germany

**Keywords:** rare species, caddisflies, Balkans, *Potamophylax winneguthi* species group, microscale endemism

## Abstract

**Background:**

The Western Balkans are an important hotspot of caddisfly diversity in Europe, with several microscale endemics, many of which were discovered during the recent years. The genus *Potamophylax* Wallengren, 1891 likely originated and diversified in Europe, with the Balkan Peninsula being one of the most important diversity hotspots.

**New information:**

In this paper, we describe the new species *Potamophylax coronavirus* sp. n. from Bjeshkët e Nemuna National Park in the Republic of Kosovo. The new species belongs to the *Potamophylax winneguthi* species group and is morphologically most similar to *Potamophylax juliani* Kumanski, 1999, currently known only from Bulgaria and *Potamophylax winneguthi* Klapalek, 1902, known from Bosnia and Herzegovina and Serbia. The male of the new species differs from its most similar congeners mainly in exhibiting: (1) elongated subrectangular superior appendages in lateral view; (2) hardly acuminate, almost rounded apex of intermediate appendages; (3) differently shaped, irregular and higher inferior appendages; (4) narrow spinate area, roughly rectangular in dorsal view, slightly wider at the base and (5) different paramere shape and/or spine pattern. The new species also differs by its considerably smaller size and association with open, high altitude eucrenal zones.

The uncorrected interspecific pairwise distance between *P. coronavirus* and other species of the *P. winneguthi* species group is on par with those amongst other recognised species in the group, as well as with the yet ambiguously identified taxa from the Sharr, Rila and Bajgorë Mountains.

The new species is most probably a microendemic of Bjeshkët e Nemuna, thus highlighting further this area as an important hotspot of caddisfly biodiversity in Europe.

## Introduction

The genus *Potamophylax* likely originated and diversified in Europe, with only seven taxa distributed outside the continent, in nearby Asia (Mey 1979, Darılmaz and Salur 2015, Sipahiler 2014). Species of this genus are frequently found in different habitats of freshwater ecosystems, such as spring areas, small brooks, large rivers and lakes as well (Graf et al. 2008). Larvae of some species have also been found in man-made habitats and brackish waters (e.g. Williams and Williams 1998). Adults are found from early spring up to late autumn, with some species undergoing a summer diapause. While many species of this genus are widely distributed, several others have a narrow area of distribution and are mostly found in the Balkan Peninsula and the Iberian Peninsula, which are probably the main speciation areas (Martinez et al. 2016, Oláh et al. 2013, Oláh and Kovács 2012).

The ongoing caddisfly investigations in the Balkan Peninsula during the past decade have discovered several new species, have increased the knowledge about the ecology of many other species and contributed to raising awareness about conservation of this order of aquatic insects and associated habitats (e.g. Ibrahimi et al. 2015, Ibrahimi et al. 2016, Ibrahimi et al. 2019a, Olah et al. 2018, Vitecek et al. 2017). For example, this increased knowledge about Balkan caddisflies has culminated in the listing of 53 species into the Red Book of Fauna of the Republic of Kosovo (Ibrahimi et al. 2019c). This is the first inclusion of Trichoptera in Red Lists in south-eastern Europe. However, there are still areas in the Balkan Peninsula which are under-investigated or not investigated at all.

Several new records and species have been reported lately from the Bjeshkët e Nemuna Mountains, from Albania and Kosovo (e.g. Ibrahimi et al. 2014, Ibrahimi et al. 2019a, Ibrahimi et al. 2019b, Malicky 1981, Malicky 1986, Oláh et al. 2017, Vitecek et al. 2015b); however, the number of caddisfly species from this mountainous massif is still low. Considering the great diversity of freshwater habitats in the Bjeshkët e Nemuna Mountains that expand into three countries (Albania, Montenegro and Kosovo), we posit that the number of caddisfly species is considerably higher than currently known.

In this paper, we describe a new species of *Potamophylax* from Bjeshkët e Nemuna in the Republic of Kosovo, with molecular and ecological notes included and also discuss morphological and ecological features of other species of the *Potamophylax winneguthi* species group.

## Materials and methods

### Fieldwork, identification and taxonomic work

**Figure 1.**
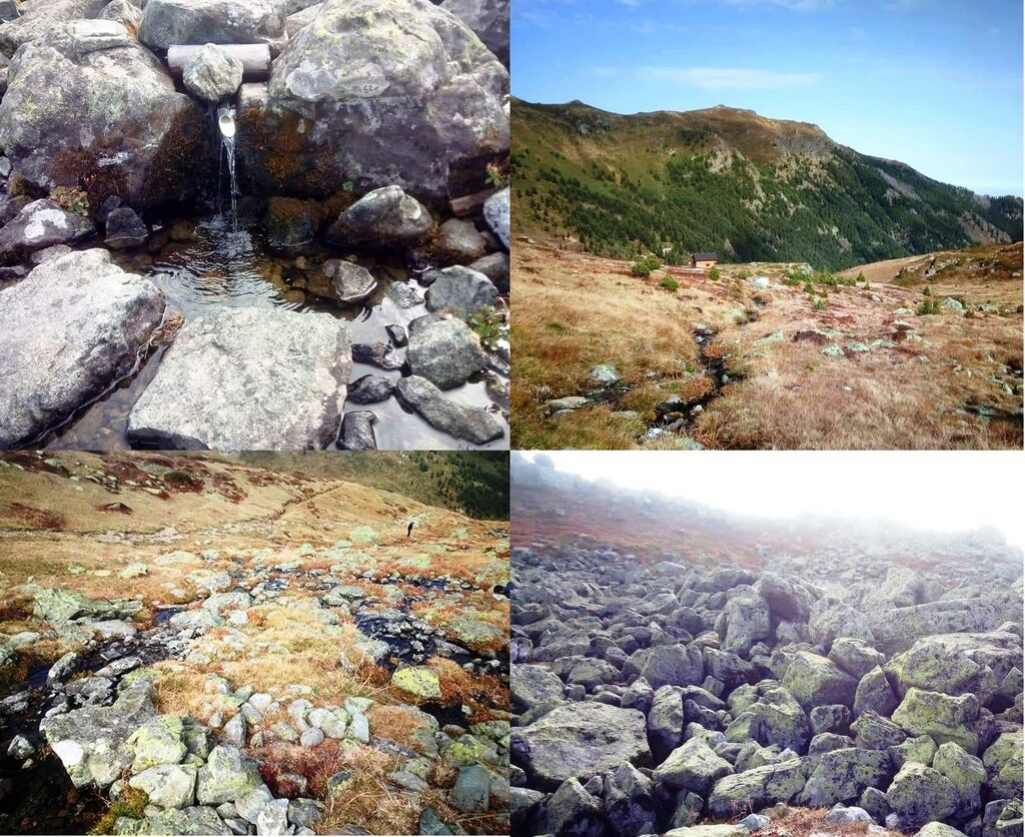
Pictures of type locality of *Potamophylax coronavirus* sp. n.: Krojet e Ali Pashë Gucisë.

We collected adult caddisflies with entomological nets and handpicking from the riparian vegetation near the streams, as well as with ultraviolet light trapping in the vicinity of the streams. Nocturnal light trapping followed Malicky (2004). Specimens were stored directly in 80% ethanol. Specimens of the new species were collected at two localities in the Lloqan Mountain, belonging to the Bjeshkët e Nemuna massif in the Deçan Municipality (Fig. 1). Specimens of three other, still unidentified, taxa of the *Potamophylax winneguthi* species group were collected in two areas in the Republic of Kosovo: Sharr Mountains and Bajgorë and Rila Mountain in Bulgaria. Details of the sampling stations and specimens are given in the examined material list further on and in Table 1. The collected material is deposited at the Department of Biology, Faculty of Mathematics and Natural Sciences, University of Prishtina “Hasan Prishtina”, Prishtinë, Republic of Kosovo. In addition, for comparative assessments of morphological features of *Potamophylax coronavirus* sp. n., we used specimens collected in Osogovo Mountain in Bulgaria (*Potamophylax juliani*) and Zlatibor Mountain in Serbia (*Potamophylax winneguthi*). For the lacking species, comparative assessment was done, based on literature (Malicky 2004, Kumanski and Malicky 1999, Oláh and Kovács 2012).

Morphological characteristics of male terminalia of the new species were examined in cleared specimens. Nomenclature of male terminalia follows Nielsen (1957), for *Limnephilus flavicornis* (Fabricius, 1787) and Kumanski and Malicky (1999). The rest of the specimens were identified using publications by Kumanski (1988), Malicky (2004) and Oláh et al. (2017). Systematic nomenclature follows Morse (2020).

Morphological features of genitalia of *Potamophylax coronavirus* sp. n. were analysed from seven male specimens. Illustrations were prepared in Adobe Illustrator (version Creative Cloud 2018) by digitising pencil templates made with a camera lucida.

### DNA extraction and PCR amplification

Whole genomic DNA was extracted from the abdomen of adult or individual legs of larval specimens using the DNEasy Blood and Tissue Kit (Qiagen, Hilden), following the manufacturer’s protocol. Three mitochondiral gene regions (mtCOI5-P, mtCOI3-P and 16S rDNA) and three nuclear gene regions (CADH, WG and 28S nrDNA) were amplified using standard PCR procedures and primers as described in Vitecek et al. (2015a). All these genes have been shown to successfully discriminate closely-related species in various Trichoptera families (e.g. Vitecek et al. 2015a, Hjalmarsson et al. 2019). Reactions for PCR amplifications were set up in 10 μl reactions. PCR products were visualised on 1.5% agarose gels and purified in the case of nuclear 28S nrDNA (Qiagen Gel Extraction; Qiagen) and sequenced in both directions on an ABI 3730XL sequencer (Applied Biosystems, Waltham) at the Senckenberg Biodiversity and Climate Research Centre, Frankfurt. In order to obtain full length information of the 1627 bp long 28S gene (D1–D3 regions), two internal primers (D2-UP-4 / D2DN-B; Zhou et al. 2007) were used.

### Sequence alignment and phylogenetic analyses

Raw ABI sequence reads were edited and merged in Geneious R6 (https://www.geneious.com). Sequences were aligned using MAFFT (Katoh and Standley 2013) and manually trimmed to exclude regions with > 80% missing data in the peripheral regions of the alignments. Phylogenetic relationships were inferred using Bayesian and Maximum-Likelihood methods, based on a concatenated alignment of 2918 bp length (Table 1). Nucleotide substitution models were selected for each method separately, using automatic substitution model selection via bModelTest (Bouckaert and Drummond 2017) and ModelFinder (Kalyaanamoorthy et al. 2017), as implemented in BEAST2 (Bouckaert et al. 2014) and IQ-TREE2 (Minh et al. 2020), respectively. For Bayesian Inference through MrBayes 3.2 (Ronquist et al. 2012), appropriate nucleotide substitution models for each partition were selected using MEGA (Stecher et al. 2020). Protein coding genes were partitioned by codon; 16S rDNA and 28S nrDNA were not. Bayesian Inference through BEAST2 to estimate phylogenetic relationships was done by running analyses of 10×10^9^ generations and sampling every 10,000^th^ generation. Bayesian Inference through MrBayes was conducted in 2 parallel runs with 2 chains each, running for10×10^6^ generations and sampling every 10,000^th^ generation. BEAST log files and MrBayes parameter files were visualised in Tracer1.6 (Rambaut et al. 2014) and checked for convergence of model parameters. Maximum-clade-credibility trees were estimated with TreeAnnotator from Bayesian tree samples obtained through BEAST2 after discarding 80% of the samples as runs failed to become stationary before this threshold. A majority rule consensus tree was estimated from the trees sampled by MrBayes after discarding the first 50% of the sample as average standard deviation of split frequencies reached values < 0.01 past that point. Finally, a Maximum-Likelihood tree was inferred using IQ-TREE2 and support values estimated, based on 1000 ultrafast bootstrap samples (Hoang et al. 2018), as well as 1000 replicates of an SH-like approximate likelihood ration test (Guindon et al. 2010).

Phylogenetic reconstruction was conducted twice with each method to assure stability of recovered tree topologies. Results of phylogenetic analyses are presented using the Maximum-Likelihood topology where node support values also include Bayesian posterior probabilities; topologies inferred through Bayesian methods are provided in the Supplementary files (Suppl. materials 1, 2).

## Taxon treatments

### Potamophylax coronavirus

Ibrahimi, Bilalli & Vitecek sp. n.

2834D389-6FE2-5DE0-98B3-53E102C1AFAD

A0208ED4-C6EC-43FB-8FED-6E16AF16DABB

#### Materials

- **Type status:** Holotype. **Occurrence:** recordedBy: Halil Ibrahimi; individualCount: 1; sex: male; lifeStage: adult; **Taxon:** class: Insecta; order: Trichoptera; family: Limnephilidae; genus: Potamophylax; specificEpithet: coronavirus; taxonRank: species; scientificNameAuthorship: Ibrahimi, Bilalli & Vitecek; nomenclaturalCode: ICZN; **Location:** continent: Europe; waterBody: Adriatic Watershed; country: Kosovo; countryCode: XKS; municipality: Deçan; locality: Bjeshkët e Nemuna, Lloqan Mountain.; verbatimLocality: A tributary of Lumbardhi i Deçanit River, Krojet e Ali Pashë Gucisë springs.; verbatimElevation: 2066; decimalLatitude: 42.5491; decimalLongitude: 20.13833; **Event:** samplingProtocol: entomological net; year: 2014; month: 10; day: 13; **Record Level:** datasetName: Trichoptera Bjeshket e Nemuna
- **Type status:** Other material. **Occurrence:** recordedBy: Halil Ibrahimi; individualCount: 1; sex: female; lifeStage: adult, with slightly damaged terminalia; **Taxon:** class: Insecta; order: Trichoptera; family: Limnephilidae; genus: Potamophylax; specificEpithet: coronavirus; taxonRank: species; scientificNameAuthorship: Ibrahimi, Bilalli & Vitecek; nomenclaturalCode: ICZN; **Location:** continent: Europe; waterBody: Adriatic Watershed; country: Kosovo; countryCode: XKS; municipality: Deçan; locality: Bjeshkët e Nemuna, Lloqan Mountain.; verbatimLocality: A tributary of Lumbardhi i Deçanit River, Krojet e Ali Pashë Gucisë springs.; verbatimElevation: 2066; decimalLatitude: 42.5491; decimalLongitude: 20.13833; **Event:** samplingProtocol: entomological net; year: 2014; month: 10; day: 13; **Record Level:** datasetName: Trichoptera Bjeshket e Nemuna
- **Type status:** Paratype. **Occurrence:** recordedBy: Halil Ibrahimi, Agim Gashi; individualCount: 2; sex: male; lifeStage: adult; **Taxon:** class: Insecta; order: Trichoptera; family: Limnephilidae; genus: Potamophylax; specificEpithet: coronavirus; taxonRank: species; scientificNameAuthorship: Ibrahimi, Bilalli & Vitecek; nomenclaturalCode: ICZN; **Location:** continent: Europe; waterBody: Adriatic Watershed; country: Kosovo; countryCode: XKS; municipality: Deçan; locality: Bjeshkët e Nemuna, Lloqan Mountain.; verbatimLocality: A tributary of Lumbardhi i Deçanit River, Gurrat e Hasan Agës springs.; verbatimElevation: 2218; decimalLatitude: 42.560696; decimalLongitude: 20.153876; **Event:** samplingProtocol: entomological net; year: 2014; month: 10; day: 15; **Record Level:** datasetName: Trichoptera Bjeshket e Nemuna
- **Type status:** Paratype. **Occurrence:** recordedBy: Halil Ibrahimi, Astrit Bilalli, Linda Grapci-Kotori, Donard Geci, Edison Kasumaj; individualCount: 4; sex: male; lifeStage: adult; **Taxon:** class: Insecta; order: Trichoptera; family: Limnephilidae; genus: Potamophylax; specificEpithet: coronavirus; taxonRank: species; scientificNameAuthorship: Ibrahimi, Bilalli & Vitecek; nomenclaturalCode: ICZN; **Location:** continent: Europe; waterBody: Adriatic Watershed; country: Kosovo; countryCode: XKS; municipality: Deçan; locality: Bjeshkët e Nemuna, Lloqan Mountain.; verbatimLocality: A tributary of Lumbardhi i Deçanit River, Krojet e Ali Pashë Gucisë springs.; verbatimElevation: 2066; decimalLatitude: 42.5491; decimalLongitude: 20.13833; **Event:** samplingProtocol: entomological net; year: 2014; month: 11; day: 21; **Record Level:** datasetName: Trichoptera Bjeshket e Nemuna
- **Type status:** Paratype. **Occurrence:** recordedBy: Halil Ibrahimi, Astrit Bilalli, Linda Grapci-Kotori, Donard Geci, Edison Kasumaj; individualCount: 2; sex: male; lifeStage: adult; **Taxon:** class: Insecta; order: Trichoptera; family: Limnephilidae; genus: Potamophylax; specificEpithet: coronavirus; taxonRank: species; scientificNameAuthorship: Ibrahimi, Bilalli & Vitecek; nomenclaturalCode: ICZN; **Location:** continent: Europe; waterBody: Adriatic Watershed; country: Kosovo; countryCode: XKS; municipality: Deçan; locality: Bjeshkët e Nemuna, Lloqan Mountain.; verbatimLocality: A tributary of Lumbardhi i Deçanit River, Krojet e Ali Pashë Gucisë springs.; verbatimElevation: 2066; decimalLatitude: 42.5491; decimalLongitude: 20.13833; **Event:** samplingProtocol: entomological net; year: 2020; month: 12; day: 03; **Record Level:** datasetName: Trichoptera Bjeshket e Nemuna

#### Description

**Figure 2.**
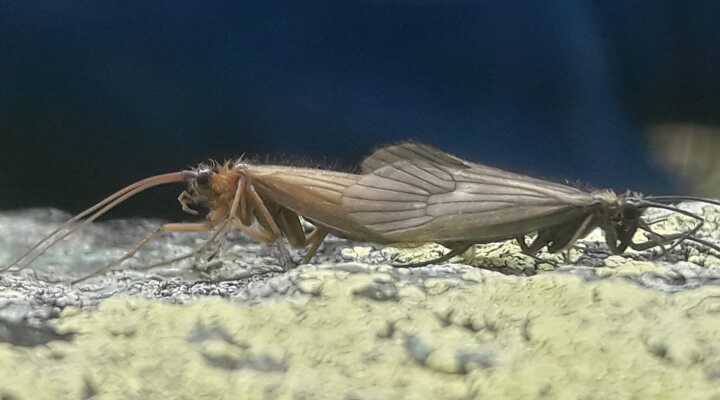
Adults of *Potamophylax coronavirus* sp. n. in copulation.

**Figure 3.**
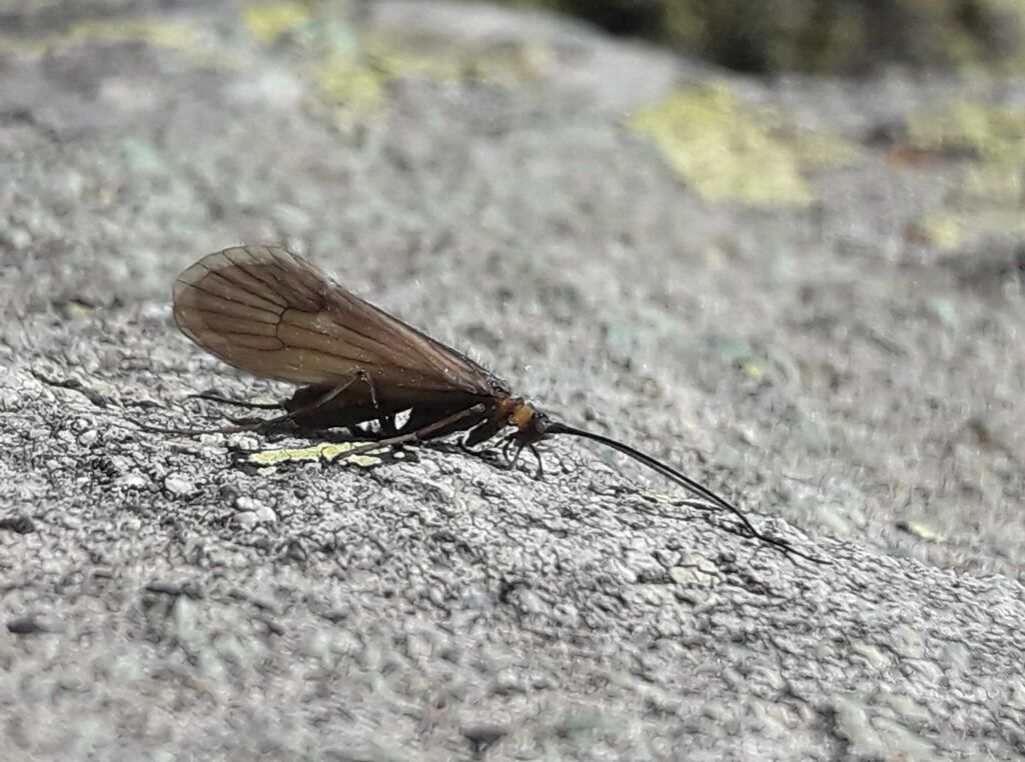
Male adult of *Potamophylax coronavirus* sp. n.

**Male.** *General appearance* (Figs 2, 3). Head and appendages brown, prothorax, sclerites of meso and metathorax and coxae dark brown to black; femora and tibiae brown, tarsi gradually darkening towards the apex. Wings dark brown with dark setae. Male maxillary palp 3 segmented. Forewing length 10 – 11.5 mm, spur formula 1-3-4.

**Figure 4.**
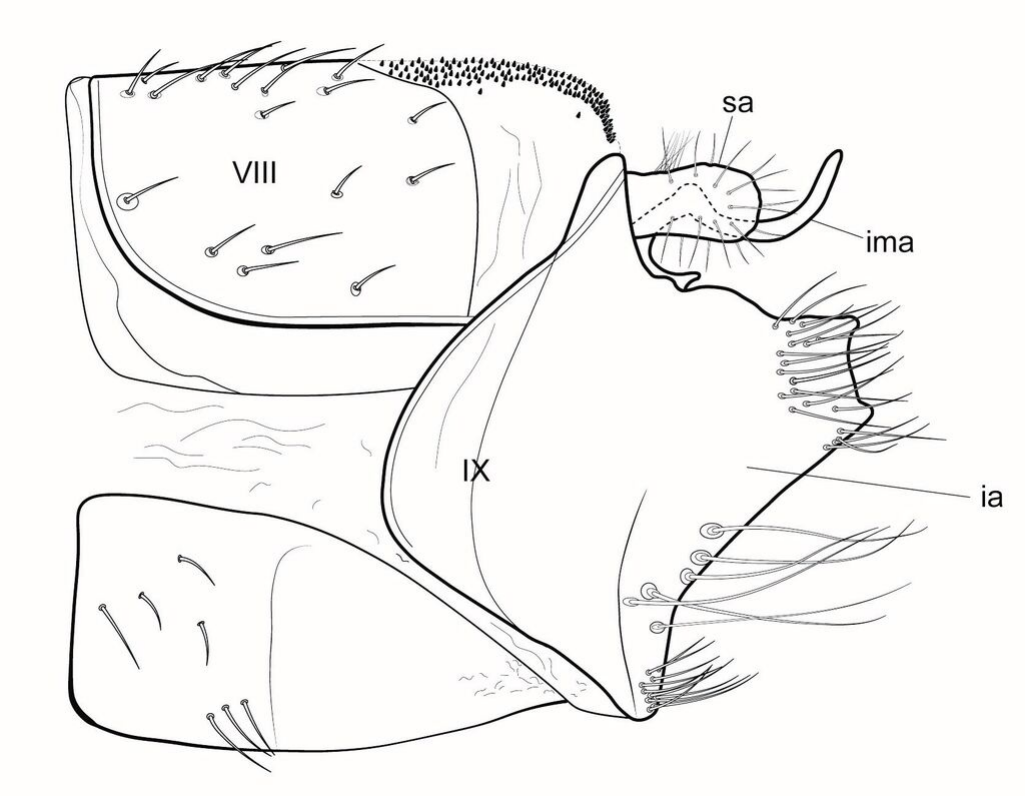
Left lateral view of the male genitalia of *Potamophylax coronavirus* sp. n. VIII - tergite VIII, IX - segment IX, ia - inferior appendages, ima - intermediate appendages, sa - superior appendages.

**Figure 5.**
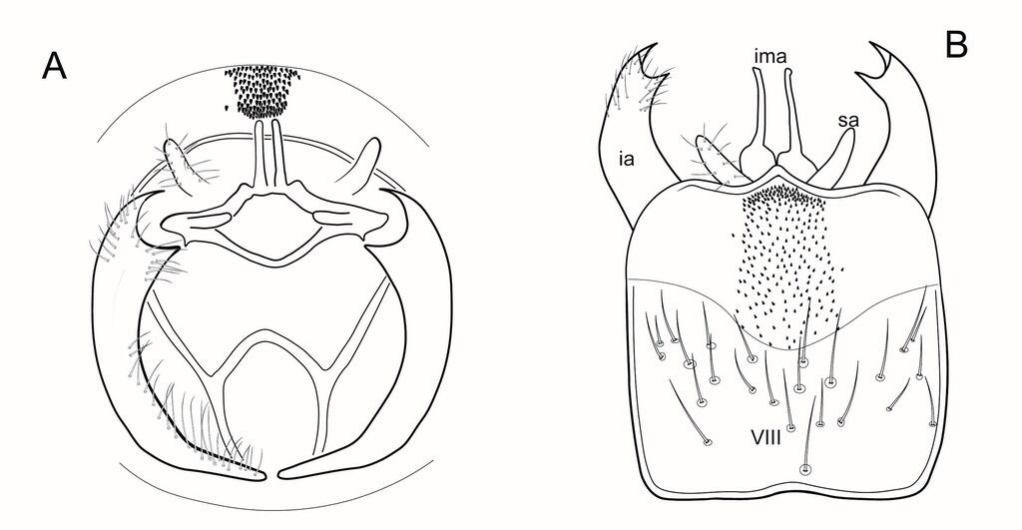
Caudal (A) and dorsal (B) view of the male genitalia of *Potamophylax coronavirus* sp. n. VIII - tergite VIII, ia - inferior appendages, ima - intermediate appendages, sa - superior appendages.

**Figure 6.**
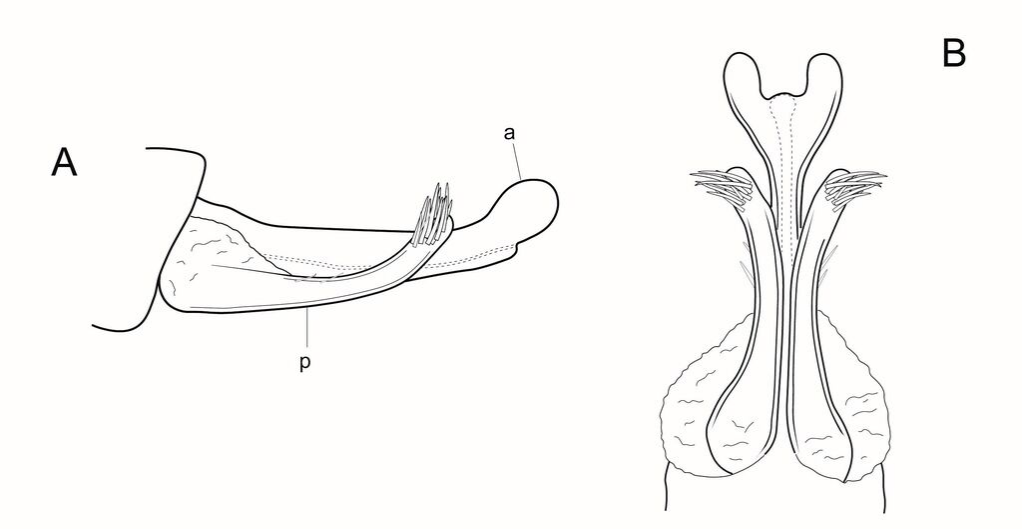
Aedeagus (a) and parameres (p) of the male genitalia of *Potamophylax coronavirus* sp. n. in lateral (A) and ventral view (B).

*Male genitalia* (Figs 4, 5, 6). Tergite VIII dark brown to black, darker than the preceding tergites, in dorsal view roughly quadratic in shape, with dorsal portion slightly narrower, posterior margin with distinct median lobe; setation concentrated on proximal portion of segment VIII, which is well sclerotised, spinate area roughly rectangular in shape with a slightly wider base in dorsal view, located on the semi-membranous distal portion of segment VIII, covered by small black spines, which are more abundant at the apex. Segment IX laterally broad, with rounded proximal areas entering into segment VIII, with short and narrow dorsal and ventral portions. Superior appendages in lateral view long, subrectangular, with rounded tips, slightly narrowing at their basal part, covered with thin setae of medium length. Intermediate appendages long, sickle-shaped with slightly rounded apex, turned upwards. Inferior appendages long, asymmetric, broadly connected with the segment IX and fused throughout most of their length, the separation line between them and segment IX visible only in basal third, bulging dorsally at the area between them and segment IX, their protruding upper portion truncated squarely, both dorsal and ventral corners of the upper portion forming rounded points, parallel to each other, directed mesally. Phallic apparatus consists of an aedeagus of medium height and a pair of parameres. Aedeagus bulbous, narrow in the middle, enlarged at the tip with bifid apex, apicomesal excision wide-U-shaped. Parameres robust, brown-black in colour, with a wider base, narrowing gradually towards the apex, with a bunch of short and very thick spines originating mainly at the distal third and a few of them ventraly.

**Female.** A single female specimen collected during the field trip generally resembles the female of *Potamophylax juliani*, but smaller in size.

Smaller and of lighter colour than the male. Head and appendages brown, prothorax, sclerites of meso and metathorax light brown to brown; femora and tibiae brown, tarsi gradually darkening towards the apex. Brachypterous. Forewings light brown in colour, shorter than abdomen, with very long and strong erect setae, mostly on the longitudinal veins. Forewing length 8.5 mm. Spur formula 1-3-4. Antennae slender.

It has slightly damaged terminalia and thus we currently refrain from describing genitalia.

#### Diagnosis

Males of the new species are most similar to *Potamophylax juliani*, currently known only from Bulgaria and *Potamophylax winneguthi*, known from Bosnia and Herzegovina and Serbia, but differ in exhibiting: (1) elongated subrectangular superior appendages in lateral view, slightly narrowing at their base, rounded at the apex; (2) hardly acuminate, almost rounded apex of intermediate appendages in lateral view; (3) differently shaped inferior appendages, bulging dorsally at the area between them and segment IX in lateral view, with high and broad upper protruding portion, with a narrow distance between dorsal and ventral corners of the upper portion, which are set parallel to each other and directed mesially; (4) spinate area narrow, roughly rectangular in dorsal view, only slightly wider at the base and (5) short stout parameres with base wider than the apex and short, very thick spines originating below the apex, only slightly reaching above the apex and few other smaller ones proximally. *Potamophylax juliani* males have: (1) small, laterally rounded, ovoid superior appendages; (2) long, slender intermediate appendages with sharply acuminate apex in lateral view; (3) rather short inferior appendages, with a shortened protruding upper portion as high as half of the entire appendage’s height, with a wide distance between dorsal and ventral corners, which are set parallel to each other and directed mesially; (4) spinate area in dorsal view narrow at the apex and almost three times wider at the base and (5) short stout parameres with very wide basal third and narrow apex, with 15 – 20 thick spines of medium length originating mostly from the tip. *Potamophylax winneguthi* males have: (1) small, laterally semicircular superior appendages; (2) long slender intermediate appendages with acuminate apex; (3) inferior appendages parallel-edged, dorsally truncated in a rectangular manner, longer on their ventral edge, directed dorsad; (4) spinate area wide, covering almost the entire width of the distal portion of segment VIII in dorsal view and (5) short stout parallel-edged parameres with almost same width along the entire length, only slightly narrower at their middle part, with 5-7 very long spines originating from distal half.

The new species also differs from both of its most similar congeners by its considerably smaller size and different type of habitat, inhabiting open high altitude eucrenal zones.

#### Etymology

The species epithet *coronavirus* relates to the *severe acute respiratory syndrome coronavirus 2 (SARS-CoV-2)* which caused a global pandemia starting 2020. The current paper was written during the quarantine time due to the pandemics. The species epithet also emphasises figuratively another silent pandemic occurring on freshwater organisms in Kosovo rivers, due to the pollution and degradation of freshwater habitats, including particularly the increased activity of mismanaged hydropower plants.

#### Distribution

During the field survey in the Bjeshkët e Nemuna Mountains, we found *Potamophylax coronavirus* sp. n. at only two localities within a 3 km perimeter, although several other springs and brooks were sampled.

#### Ecology

Both sampling stations are open spring areas, located above 2000 m a.s.l. The substrate of streams close to the sampling sites was dominated by meso- to macrolithal, surrounded by riparian vegetation. The species was collected during the day by handpicking and entomological nets. The species was not observed flying, implying low flying activity. No specimen was caught with light traps. The species was collected during late September, October, November and early December, implying it has an autumn flying period. In both sites, the specimens of the new species were collected only within a one-kilometre perimeter from the spring area downwards along the stream, implying that it is a typical eucrenal species.

In both sampling stations, *Potamophylax coronavirus* sp. n. was found in sympatry with the following species: *Rhyacophila tristis* Pictet, 1834, *Allogamus uncatus* (Brauer, 1857), *Drusus botosaneanui* Kumanski, 1968, *Drusus krusniki* Malicky, 1981 and *Drusus fortos* Ibrahimi and Oláh, 2017.

#### Results of phylogenetic analysis

**Figure 7.**
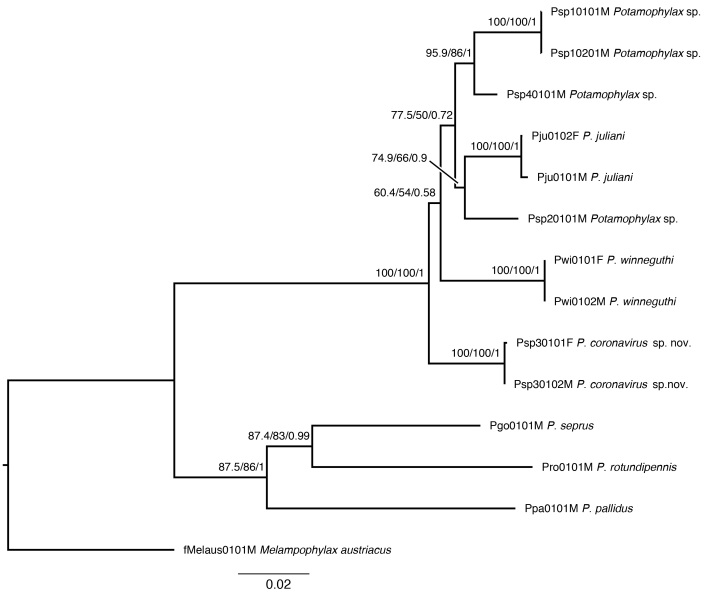
Maximum Likelihood tree of phylogenetic relationships within the *Potamophylax winneguthi* species group and closely related congeners. The *P. winneguthi* group comprises a highly supported clade with new species *P. coronavirus* sp. n. supported as sister to other species of the group, including yet ambiguously identified taxa from the Sharr, Rila and Bajgorë Mountains. Support from SH-like approximate likelihood ratio tests/Ultrafast Bootstrap/Bayesian posterior probabilities are presented above nodes; outgroup: *Melampophylax austriacus*.

Both methods recovered the same supported topology and highly similar support values. The phylogenetic analyses reveal that the *P. winneguthi* species group is monophyletic with regard to the other included *Potamophylax* species and *Melampophylax* (Fig. 7, Suppl. materials 1, 2). Within the *P. winneguthi* species group, relationships are largely unresolved. Only the proposed conspecificity of males and females is confirmed in all four species (posterior probabilities of 1.0, high bootstrap support). In addition, the *Potamophylax* spp. from Kosovo and Bulgaria appear to have a sister relationship in this group. The uncorrected interspecific pairwise distance between *P. coronavirus* and other species of the *winneguthi* species group is on par with those amongst other recognised species in the group, as well as with the yet ambiguously identified taxa from the Sharr, Rila and Bajgorë Mountains (0.03-0.05;Suppl. materials 2, 3).

## Discussion

*Potamophylax coronavirus* sp. n. is a member of the *Potamophylax winneguthi* species group. This group contains the following species: *Potamophylax winneguthi*, *Potamophylax haidukorum* Malicky, 1999, *Potamophylax juliani*, *Potamophylax gurunaki* Malicky, 1992, *Potamophylax alsos* Oláh, 2014, *Potamophylax hajlos* Oláh, 2012, *Potamophylax kesken* Oláh, 2012, *Potamophylax tagas* Oláh & Kovacs, 2012 and *Potamophylax coronavirus* sp. n. All of them are restricted to certain areas in the Balkan Peninsula: *P. winneguthi* in Varosh and some nearby localities around Sarajevo (Bosnia and Herzegovina) and Zlatibor (Serbia), *P. haidukorum* nearby Hajducka Voda (Bosnia and Hercegovina), *P. juliani* in Osogovo Mountain (Bulgaria), *P. gurunaki* in Vernon (Greece), *P. alsos* in Jabllanica (North Macedonia), *P. hajlos* in Mali i Gropës (Albania) and *P. kesken* and *P. tagas* in Korab (Albania) (Kumanski and Malicky 1999, Oláh and Kovács 2015).

Morphologically, *Potamophylax coronavirus* sp. n. forms a cluster together with *Potamophylax winneguthi*, *Potamophylax haidukorum* and *Potamophylax juliani*. This cluster is characterised by a bilobed apical margin of inferior appendages in lateral and caudal views. In this cluster, *Potamophylax coronavirus* sp. n. is small (male wing length 10-11.5 mm), while the other species of the group have wing lengths from 11-18 mm. The other cluster of this species group, the *Potamophylax tagas* species cluster, comprises *P. alsos*, *P. hajlos*, *P. kesken* and *P. tagas* and is characterised by a rounded apical margin of the inferior appendages without any significant projection. The remaining species of this species group, *Potamophylax gurunaki* is characterised by its remarkable pointed long apicodorsal corner of inferior appendages and longer rounded apicoventral corner in lateral view.

During this investigation in Kosovo, we sampled specimens of two other taxa closely resembling *Potamophylax juliani*: one from the Bajgorë area in northern Kosovo and the second one from the Sharr Mountains in western Kosovo. The comparison of these specimens with the specimens of *Potamophylax juliani* from the type locality showed that there are certain differences, mostly in the shape of inferior appendages and phallic apparatus. In *Potamophylax juliani*, the ventral margin of inferior appendages is sharply angled at its distal third, while, in the taxon from Bajgorë area, the ventral margin is curved, but not sharply angled and in the taxon from the Sharr Mountain, it is almost concave. The aedeagus, parameres and superior appendages of both populations have also certain differences from those of *P. juliani*. The male specimen collected in Rila Mountian in Bulgaria differs from *P. juliani* too by the shape of inferior appendages and width of parameres. However, the number of specimens examined in all these three taxa is too small to draw a conclusion related to the morphology of these populations. More specimens from these areas are needed to show if these differences are stable and consequently to resolve their taxonomic position.

The phenology of *Potamophylax coronavirus* sp. n. is similar to the other species of the *Potamophylax winneguthi* species group. All of them are typical autumn species. However, unlike other species of this group, specimens of *P. coronavirus* sp. n. were also collected during early December in 2018, when the daily temperatures were above 13ºC. Habitat requirements of the new species are different from all other species of the *Potamophylax juliani* species cluster collected throughout Balkans. All other species inhabit mostly spring areas or rivers inside the forested areas, while the new species was only found at open spring areas, well above the tree line. Similar to the other species of the group, the single female *Potamophylax coronavirus* sp. n. specimen collected during this investigation was brachypterous and unable to fly.

Freshwater ecosystems in the area where the new species was found, have been extremely threatened during the past years by anthropogenic activities, such as deforestation, habitat destruction, hydropower plants and touristic activities. In addition, many springs in the area are mismanaged and endangered by individual water intake pipes from nearby houses and touristic facilities. The change in water regime may greatly threaten aquatic diversity in the near future. For example, the downstream segments of the Lumbardhi i Deçanit River, whose tributaries are springs where the new species was found, were severely deteriorated during the past years due to the construction of a hydropower plant. The prescribed minimal flows are not implemented in practice and most of the time are too low to sustain aquatic biota.

The description of a new micro-endemic *Potamophylax* species from this area documents that the species of *Potamophylax winneguthi* species group are restricted to isolated highland freshwater habitats, similar to some species of the genera *Drusus* Stephens, 1837 and *Chaetopteroides* Kumanski, 1987. The high number of micro-endemics in *Potamophylax* may also be the result of a combination of geological processes and low dispersal capacity, as hypothesised for the Balkan *Drusus* species (Previšić et al. 2014). Very often, species of *Potamophylax winneguthi* species group live in sympatry with species of these two genera, although unlike Drusinae, species of this species group were found also in warmer and smaller springs and streams of lower altitudes. This investigation highlights the Bjeshkët e Nemuna Mountains as an important area, which harbours rare species of caddisflies, which are, at the same time, critically endangered by ongoing activities causing the degradation of freshwater habitats.

## Supplementary Material

676F1025-E2E6-5F70-8E6B-7F940FB9A3D3 10.3897/BDJ.9.e64486.suppl1 Supplementary material 1 Maximum clade credibility tree of phylogenetic relationships within the *Potamophylax winneguthi* species group and closely related groups inferred from one of two Bayesian tree samples obtained via BEAST. The new species *P. coronavirus* sp. n. is supported as sister to other taxa of the highly supported *P. winneguthi* group. Bayesian posterior probabilites are presented next to nodes; outgroup: *Melampophylax austriacus*. Data type Genomic File: oo_524375.jpg https://binary.pensoft.net/file/524375 Halil Ibrahimi, Astrit Bilalli, Simon Vitecek, Steffen U. Pauls, Felicitas Erzinger, Agim Gashi, Linda Grapci-Kotori, Donard Geci, Milaim Musliu and Edison Kasumaj

729E2BE3-70C1-5C86-9533-60C45E140134 10.3897/BDJ.9.e64486.suppl2 Supplementary material 2 Uncorrected pairwise distances amongst study specimens on the basis of the concatenated alignment of all six gene fragments. Highlighted in grey are species and specimens from the *P. winneguthi*-complex. In the matrix, light grey fields indicate intraspecific distances; dark grey fields interspecific distances.*Values in specimen are out of range, because the CAD locus was completely missing and coded in Ns. Data type Genomic File: oo_527218.pdf https://binary.pensoft.net/file/527218 Halil Ibrahimi, Astrit Bilalli, Simon Vitecek, Steffen U. Pauls, Felicitas Erzinger, Agim Gashi, Linda Grapci-Kotori, Donard Geci, Milaim Musliu and Edison Kasumaj

00CC1C76-641C-5018-953A-E296E643F504 10.3897/BDJ.9.e64486.suppl3 Supplementary material 3 Majority rule consensus tree of phylogenetic relationships within the *Potamophylax winneguthi* species group and closely related groups inferred from two Bayesian tree samples obtained via MrBayes. The new species *P. coronavirus* sp. n. is supported as sister to other taxa of the highly supported *P. winneguthi* group. Bayesian posterior probabilites are presented next to nodes; outgroup: *Melampophylax austriacus*. Data type Genomic File: oo_527154.jpg https://binary.pensoft.net/file/527154 Halil Ibrahimi, Astrit Bilalli, Simon Vitecek, Steffen U. Pauls, Felicitas Erzinger, Agim Gashi, Linda Grapci-Kotori, Donard Geci, Milaim Musliu and Edison Kasumaj

XML Treatment for Potamophylax coronavirus

## Figures and Tables

**Table 1.** Collection data, specimen IDs and BOLD IDs of specimens used in phylogenetic analysis. Abbreviations used: HI – Halil Ibrahimi, AB – Astrit Bilalli, AG – Agm Gashi, LGK – Linda Grapci-Kotori, EK – Edison Kasumaj, DG – Donard Geci, MM – Milaim Musliu, WG – Wolfram Graf, JK - Jenõ Kontschán, DM - Dávid Murányi, TS - Tímea Szederjesi, AE - Á. Ecsedi, TK - T. Kovács, GP - G. Puskás, GM - G. Magos.

**Specimen ID**	**BOLD ID**	**Collectors**	**Collection date**	**Latitude (N)**	**Longitude (E)**	**Altitude** **m**	**Taxon**
**Pgo0101M**	SPLIM298-21	HI, AG, EK	12.08.2014	42.5518	20.1624	1666	*Potamophylax seprus*
**Psp40101M**	SPLIM299-21	ÁE, TK, GP	07.10.2011	42.139028	23.46452	1935	*Potamophylax* sp. (Rila, Bulgaria)
**Psp30101F**	SPLIM300-21	HI, AB	13.10.2014	42.549100	20.138330	2066	*Potamophylax coronavirus* sp. n.
**Psp30102M**	SPLIM301-21	HI	13.10.2014	42.549100	20.138330	2066	*Potamophylax coronavirus* sp. n.
**Pju0101M**	SPLIM302-21	JK, DM, TS	23.10.2013	42.174383	22.63443	1520	*Potamophylax juliani*
**Pju0102F**	SPLIM303-21	JK, DM, TS	23.10.2013	42.174383	22.63443	1520	*Potamophylax juliani*
**Psp20101M**	SPLIM304-21	HI, DG	29.10.2013	42.979	21.0509	1262	*Potamophylax* sp. (Bajgorë, Kosovo)
**Psp10101M**	SPLIM305-21	HI	11.11.2012	42.17506	20.97593	1410	*Potamophylax* sp. (Sharr, Kosovo)
**Psp10201M**	SPLIM306-21	HI	18.10.2012	42.17506	20.97593	1410	*Potamophylax* sp. (Sharr, Kosovo)
**Ppa0101M**	SPLIM307-21	HI, MM	21.08.2013	42.5185	20.9788	721	*Potamophylax pallidus*
**Pro0101M**	SPLIM308-21	HI, LGK	01.10.2013	42.5185	20.9788	721	*Potamophylax rotundipennis*
**Pwi0101F**	SPLIM309-21	TK, GM	03.11.2011	43.63127	19.77166	1150	*Potamophylax winneguthi*
**Pwi0102M**	SPLIM310-21	TK, GM	03.11.2011	43.63127	19.77166	1150	*Potamophylax winneguthi*
**fMelaus0101M**	SPDRU496-14	WG	20.10.2013	46.8106	14.9931	N.A.	*Melampophylax austriacus*
