# Supplementary figures and images for: Potamophylax coronavirus sp. n. (Trichoptera: Limnephilidae), a new species from Bjeshkët e Nemuna National Park in the Republic of Kosovo, with molecular and ecological notes
Source: Biodivers Data J. 2021 Apr 7;9:e64486. doi: 10.3897/BDJ.9.e64486 (PMC8046748; doi:10.3897/BDJ.9.e64486)

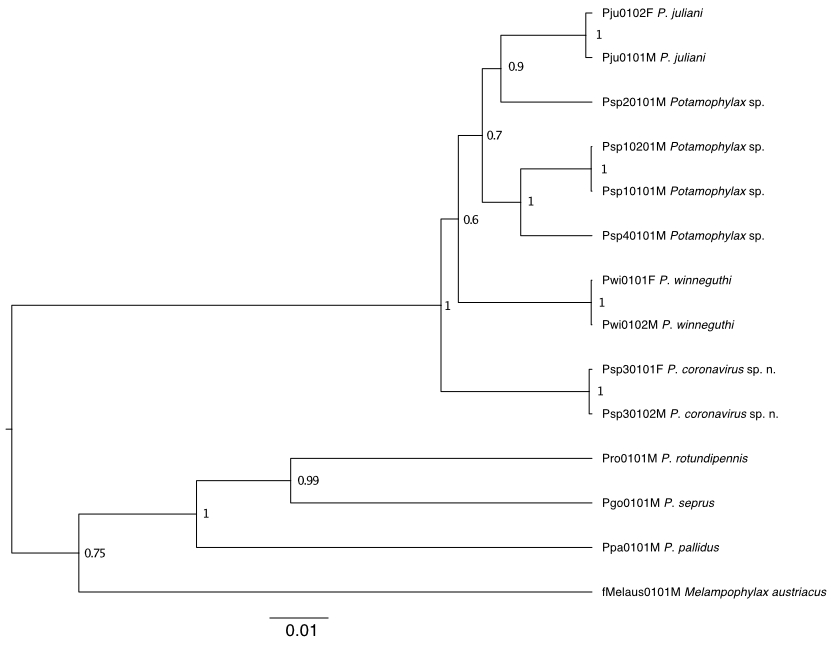

Supplement: Supplementary material 1 — Maximum clade credibility tree of phylogenetic relationships within the Potamophylax winneguthi species group and closely related groups inferred from one of two Bayesian tree samples obtained via BEAST. The new species P. coronavirus sp. n. is supported as sister to other taxa of the highly supported P. winneguthi group. Bayesian posterior probabilites are presented next to nodes; outgroup: Melampophylax austriacus. [file bdj-09-e64486-s001.jpg]

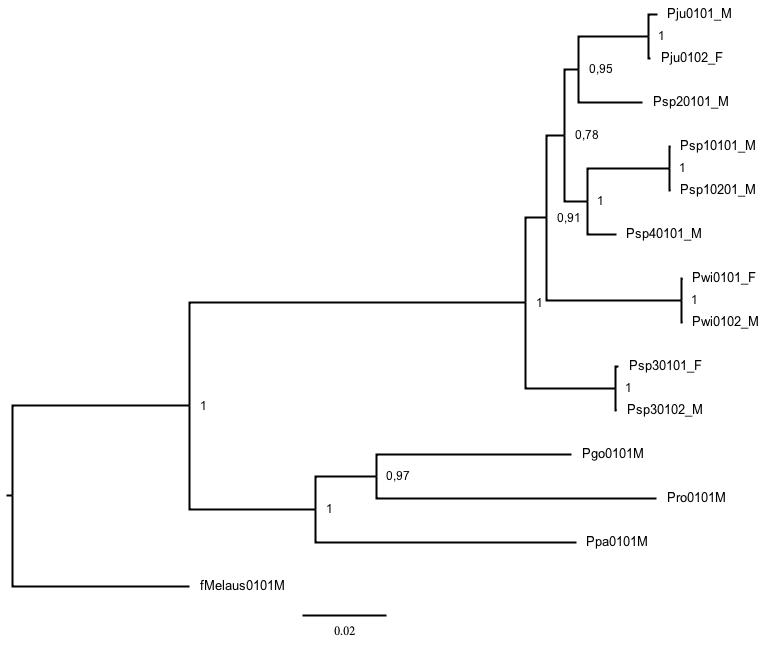

Supplement: Supplementary material 3 — Majority rule consensus tree of phylogenetic relationships within the Potamophylax winneguthi species group and closely related groups inferred from two Bayesian tree samples obtained via MrBayes. The new species P. coronavirus sp. n. is supported as sister to other taxa of the highly supported P. winneguthi group. Bayesian posterior probabilites are presented next to nodes; outgroup: Melampophylax austriacus. [file bdj-09-e64486-s003.jpg]
